# Defining and Clarifying the Terms Canine *Possessive Aggression* and *Resource Guarding*: A Study of Expert Opinion

**DOI:** 10.3389/fvets.2018.00115

**Published:** 2018-06-11

**Authors:** Jacquelyn A. Jacobs, Jason B. Coe, Tina M. Widowski, David L. Pearl, Lee Niel

**Affiliations:** ^1^Department of Population Medicine, Ontario Veterinary College, University of Guelph, Guelph, ON, Canada; ^2^Department of Animal Biosciences, University of Guelph, Guelph, ON, Canada

**Keywords:** canine aggression, possessive aggression, resource guarding, behavior, expert opinion, content analysis

## Abstract

The terms *possessive aggression* and *resource guarding* are often used interchangeably to describe behavior patterns used by a dog to control primary access to a perceived valuable item. The use of inconsistent terminology may impact the effectiveness of communication between dog owners and clinicians, affect treatment and management success for the behavior, and inhibit research progress. The aim of this study was to explore the opinions of canine behavior experts on the meaning of and preference for the terms *possessive aggression* and *resource guarding*, as well as to develop and propose an operational ethological definition for the preferential term identified. Eighty-five individuals met the inclusion criteria and were invited to participate in a two-stage online survey. Results from the two-stage survey found that the majority of participants preferred the term *resource guarding*. Detailed exploration of meaning and definitions required in-depth discussion beyond traditional survey methods, therefore, respondents from the second stage of the survey were invited to participate in an online discussion board. Following content analysis of the data from the discussion board, we conclude that the majority of participants preferred the term *resource guarding*. Considering 100% consensus was not reached regarding terminology among experts in the field, future authors and clinicians should provide clear definitions where terms are applied to ensure effective communication between all parties and to ensure consistency in canine behavior research. Based on expert contributions, we define *resource guarding* as “the use of avoidance, threatening, or aggressive behaviors by a dog to retain control of food or non-food items in the presence of a person or other animal.”

## Introduction

Canine aggression is the behavior problem most commonly referred to companion animal specialists (1), at least in part due to the potential danger involved in living with an aggressive dog and the advanced knowledge typically needed to address the problem. Treatment and management advice differs between categories of aggression (2, 3) and as such, it is important for clinicians to diagnose the type of aggression correctly. Clear descriptions of the behavior patterns involved are essential to proper diagnosis, and clinicians often must rely on pet owners to provide this information (2, 3). However, pet owners may struggle to effectively relay this information, given that the language used to describe behavior between lay people and clinicians may be different (4, 5). Furthermore, the use of inconsistent terminology has been recognized as a significant impediment to advancing behavioral science (6, 7). Research efforts focused on causation or efficacy of treatments for aggression may be negatively impacted when the type of aggression is misidentified or poorly described.

The terms *possessive aggression, resource guarding*, and *food-related aggression* have all been used to refer to a sequence of behavior patterns that dogs exhibit to gain or maintain primary access to a perceived valuable item when another animal or person approaches [e.g., (8, 9)]. It is unclear if these terms are considered to be synonyms or if there are fundamental differences between the terms that are not explicitly stated in the literature.

*Possessive aggression* is used frequently in scientific literature to describe a category of aggression involved in defense of a resource [e.g., (3, 10, 11)]. The descriptions in these sources share several common and distinct components. For example, Horwitz and Neilson (10) describe *possessive aggression* as “dogs that aggressively guard things (food bowl, rawhides, real bones, stolen, or found items), or objects (e.g., toys, stolen objects).” Horwitz and Neilson (12) describe *possessive aggression* when “the dog barks, growls, lunges, snaps and or bites when a person or animal approaches it while it is in possession of or near something it does not want to relinquish.” The former quote is specific regarding the objects of apparent interest, whereas the latter quote focuses on the specific behaviors involved in the overall sequence. Both sources seem to agree that the behavior involves aggression around something of perceived value for the dog. Overall (3) separates *possessive aggression* and *food-related aggression*, distinguishing between the two categories by item of apparent interest (e.g., food or non-food object). *Possessive aggression* is described as “aggression (threat/challenge/contest) that is consistently directed toward another individual who approaches or attempts to obtain a non-food object or toy that the aggressor possesses or to which the aggressor controls access.” The description for *food-related aggression* differs by the behavior being exclusively executed in the presence of something edible (“dog food, bones, rawhides, biscuits, blood, treats, or table scraps”). It is of interest that the author remarks that *food-related* and *possessive aggression* should not be considered “under the umbrella term of *resource guarding*;” the author states that this term is deleterious and prevents veterinarians and clients from evaluating the degree of abnormality of the behavior and inhibits a discussion on risk assessment for future aggression (3).

Trends in the scientific literature may not reflect general consensus or everyday application of preferred terminology. Comments regarding the term *resource guarding* in Overall (3) may be a recognition of the inconsistent application of the two terms or changing preferences in the dog owner community. Luescher and Reisner (1) refer to the behavior pattern as *resource guarding* (*possessive aggression*) in their text without a clear description, suggesting an interchangeable application between the two terms. An updated edition of Landsberg et al. (12) refers to the behavior pattern as *resource guarding* and includes the original description of *possessive aggression* with the addition of “tense” posture in the list of specified behaviors (12). Furthermore, the term *resource guarding* seems to be gaining colloquial popularity and is used almost exclusively in these types of settings, such as blogs [e.g., (13)]. The lack of standardized terminology and descriptions coupled with the complexity of the behavior generates potential issues for dog specialists, owners, and researchers.

Although a number of editorials have suggested that discussion and consensus on terminology related to companion animal behavior is needed (6, 7, 14, 15), to date there has been little research on this topic. To explore and develop an understanding of preferences for terminology and definitions among behavior specialists, an interactive approach is required where these professionals can share and discuss their perspectives and examine whether consensus is possible given commonalities and differences. Several methods can be used to achieve such understanding, such as online discussion boards, where participants can openly share their views, opinions, and ideas while remaining anonymous to each other. Discussion boards are similar to focus groups in that they are facilitated by a moderator and data are generated through participant discussion (16). The extracted data can then be analyzed using qualitative methodology, which utilize the participants' words in the context of the discussion to gain a deeper understanding of the topic.

The objective of this study was to clarify terminology and develop a definition surrounding the behavior(s) commonly referred to as *resource guarding, possessive aggression*, and *food-related aggression*. More specifically, the authors sought to determine whether canine behavior specialists felt the terms described different behaviors or were interchangeable. If participants expressed that the terms were interchangeable, the authors sought to determine if a preferential term existed and to explore reasons why the chosen term may be preferred. Additionally, the authors sought to develop and propose an acceptable ethological definition, broadly focused on animal behavior and allowing for inclusion of emotional and motivational states, for the preferential term based on considerations from participants.

An external and long-term objective of this research is to encourage further discussions surrounding the various uses of terminology and definitions for similar topics which lack clarity.

## Materials and methods

All procedures were submitted and approved by the University of Guelph Research Ethics Board prior to the start of this study.

### Participants

Experts were identified as having an advanced degree [either Doctor of Veterinary Medicine (DVM) or Doctor of Philosophy (PhD) in a related field] with additional professional requirements that indicated an advanced knowledge of companion animal behavior [i.e., Diplomat of the American College of Veterinary Behavior (DACVB), Diplomat of the European College of Animal Welfare and Behavior Medicine—Companion Animal (DECAWBM-CA), Certified Applied Animal Behaviorist (CAAB), or Certified Clinical Animal Behaviorist (CCAB)]. Eighty-five experts met the inclusion criteria at the start of the study, and were invited to participate in all stages of the survey. Thirty-six individuals participated in the first survey stage for a response of 45%. Responses from the first stage were returned to all responding participants for comment during the second stage. Twenty-nine of the thirty-six individuals participated in the second stage of the survey for a response of 80%. The majority of participants (28/29) resided in the United States or Canada. Following the second stage of the survey, the authors determined that developing a definition and understanding the terminology preferences required in-depth discussion with experts that extended beyond the ability of traditional survey techniques. Twenty-nine experts participating in the previous two stages of the survey were invited to participate in an online discussion board to provide greater opportunity for open dialogue. Fourteen individuals participated in the online discussion for a response of 48%. The majority of participants (13/14) resided in the United States or Canada. Table 1 contains educational demographics for participants in each stage of the survey and discussion board.

**Table 1:** Educational demographics of participants in each stage of the survey and discussion board.

**Stage**	**Education demographics**	**# Participants**
Survey stage 1 (85 invited)	DVM + DACVB or DECVBM-CA	21
	DVM + DACVB & CAAB	3
	PhD + CAAB or CCAB	12
	Total	36/85 (45%)
Survey stage 2 (36 invited)	DVM + DACVB or DECVBM-CA	15
	DVM + DACVB & CAAB	2
	PhD + CAAB or CCAB	12
	Total	29/36 (80%)
Discussion board (29 invited)	DVM + DACVB or DECVBM-CA	7
	DVM + DACVB & CAAB	2
	PhD + CAAB or CCAB	5
	Total	14/29 (48%)

### Procedure

Initially, a two-stage survey was employed to gather expert opinion regarding concepts, definitions, treatment, and prevention methods for the behavioral response(s) commonly referred to as *resource guarding, possessive aggression*, or *food-related aggression*. The current paper is focused solely on concepts and definitions. For the first survey stage, all identified participants were invited in January, 2013 through an introductory email outlining the project's objectives and the format requiring multiple stages of participation. The email provided a link to an online survey website (LimeSurvey) where participants were asked several open-ended questions: (1) “How do you define resource guarding/possessive aggression?” (2) “Please outline any behavior or other factors you might use to identify resource guarding/possessive aggression. How might you differentiate this behavior from other types of aggression?” and (3) “Briefly outline the treatment methods you would recommend to an owner of a dog with resource guarding/possessive aggression.”

All participants that completed the first survey stage were invited to participate in the second survey stage in March, 2013, which included closed- and open-ended questions that were developed from the results of content analysis on the first stage's responses (see below for further detail on methods). Thus, the content in the second survey was participant driven and provided opportunity for participants to anonymously comment on responses from the first stage (17). Further, an open-ended clarifying question was added to the second stage to address perceived confusion in providing a definition for the behavior: “Do you prefer the term resource guarding, possessive aggression, or a different term and why?”

Following the first two survey stages, the authors determined that identifying a universally accepted definition as well as exploring reasons for terminology preferences would require in-depth, dynamic discussion between experts that extended beyond the ability of traditional survey techniques. Therefore, participants from the second stage were invited to participate in an online discussion board website (20|20 research, Qualboard). After completing the consent form and initial sign-in process, a unique identifier was automatically assigned to each participant. This identifier provided them with anonymity during the online discussion board in order to encourage honest responses and reduce the potential influence of group pressure (17). The discussion board initially consisted of two questions related to terminology: (1) “Do you think the terms *possessive aggression* and *resource guarding* describe different behaviors or are they appropriate to use interchangeably? Please explain,” and (2) “What are your thoughts on the use of separate terminology when referring to this behavior when associated with food only?” Further, the authors considered all responses and comments on a definition from the first and second survey stages and derived the following definition based on a combination of the most common components proposed: “A dog that is displaying defensive, threatening, or aggressive behaviors to prevent a person or other animal from gaining access to a food or non-food object.” Participants were provided with this definition in the online discussion and asked (3) to comment on components of the definition they would like to have changed, or provide their own complete definitions. All three questions were released simultaneously to participants at the beginning of the 1 week study period (September 3rd through 10th, 2013). The discussion board was open for 7 days during which time participants were encouraged to post comments on their own schedule. They could either respond directly to the moderator's questions, or post responses to other individual's comments.

In addition to the three initial questions posed by the moderator, one probing question was added on day 3 during the online discussion to explore perceived variability in participants' definitions of aggression. Participants were asked “How do you define the word ‘aggression’? Please consider specific behaviors in your response.” On day 6 of the discussion board, the main points of the discussion were summarized by the moderator and presented back to all participants for a 24-h period in order to provide a final opportunity for clarification, confirmation of the discussion or any final thoughts from participants. The final completed discussion board was downloaded in text documents for analysis.

### Data analysis

As results from stage one were incorporated into the questions for stage two and thus provide no additional information, only results from stage two and the discussion board are presented in this paper. For stage two of the survey, we determined the number of participants that preferred to use the terms resource guarding, possessive aggression, or an alternative. A Fischer's exact test (SAS 9.4, Cary, NC) was performed to determine if there was a significant difference between expert background and preference for terminology choice. Qualitative data management software was utilized for the remainder of the data analysis (ATLAS.ti, Berlin, Germany). To understand the data and address the research questions, a qualitative approach was taken. Data was analyzed descriptively using manifest content analysis (18–20). Content analysis identifies, counts and attempts to understand the context in which specific words or ideas are expressed within the data (21–23). In this way, content analysis permits both a qualitative and quantitative approach to data analysis; descriptive coding of the data follows a quantitative count of the codes (20). Specifically, data were coded by reading the text of the open-ended responses from the survey and discussion board several times to become familiar with the data. Through an iterative process of repeated review of the text, related data were assigned key words or phrases (i.e., codes) which were then subjected to quantitative counts of the number of participants that stated or supported a particular thought or view, and supportive examples were extracted from the text.

## Results

### Terminology preference among participants

The majority of participants in stage two of the survey preferred the term “*resource guarding”* (Table 2). No significant difference was found between expert backgrounds (i.e., CAAB/CCAB vs. DACVB/DECAWBM-CA) and preference for terminology (*p* = 0.56). In the discussion board, eleven (out of fourteen) participants thought the terms *resource guarding* and *possessive aggression* described different behaviors, with the majority of these people (seven out of eleven) further expressing the belief that *possessive aggression* described a more specific version of the behavior and is only applicable when aggressive behaviors are involved. Two of the 11 individuals mentioned, unprompted, that they recognize the terms are often used interchangeably but they do not consider them to be synonyms. Two participants (out of 14) consider the terms to be synonyms and thus consider it appropriate to use the terms interchangeably. One participant did not provide a direct response to this topic.

**Table 2:** Count of terminology preference from participants in survey stage 2.

**Preferred term**	**# Participants**
Resource guarding	19/29 (66%)
Possessive aggression	6/29 (21%)
Resource guarding or possessive aggression	2/29 (7%)
Neither	1/29 (3%)

When asked about the use of separate terminology for the behavior pattern when associated with food only, the majority of participants agreed the use of a separate term is unnecessary. A few participants stated they prefer not to complicate terminology further by using a separate term specific to one type of resource. Several other participants stated that they believe the motivation remains the same regardless of the type of item being “guarded” and therefore the use of separate terminology is not meaningful. Three participants discussed that dogs may consider items other than what we consider to be food edible (such as a paper towel) and thus, it is inappropriate to ascribe a specific term when assumptions are being made about the item category. A little more than half (eight out of fourteen) of the respondents mentioned that they would use a subheading with their preferred term to describe the item or context more specifically for treatment and management purposes, but that using a completely separate term was unnecessary. Only two participants thought it was important to use separate terminology when describing *food-related aggression*. In support of this view, these two participants referenced published literature suggesting the existence of separate brain pathways mediating aggression around food compared to other objects [example provided: (24)].

### Reasons for terminology preference

#### The influence and meaning of the word “aggression”

Eleven (out of 14) respondents suggested that the word “aggression” within the term *possessive aggression* has an influence on the meaning and application of the term and describes a behavioral sequence that necessitates an aggressive response. Specifically, these participants stated that *possessive aggression* was not a broad enough term to include the behaviors of “body blocking” or “grabbing an item and running away with it.” For example, one respondent stated

“*I always use resource guarding, which is a broader term than possessive aggression. A dog may in fact guard just by staring and/or body blocking without showing an aggressive response.”*

Respondents considered the inclusion of the word “aggression” to limit the referenced behavior to the aggressive form, and for some, the use of the word “aggression” in the term implied a more severe form of the behavior. For example, one respondent stated

“*…some animals will guard the resource without overt aggressive responses but will freeze and hover. I tend to use both terms…but when the dog becomes more threatening I may use possessive aggression but at times have also used resource guarding aggression.”*

Three respondents (out of 14) disagreed with other participants' rationale for distinguishing between terms with and without the word “aggression,” stating that inclusion of this word should not be a limiting factor for the meaning of the term as aggression can include a range of behaviors not limited to snapping or biting. For example, one respondent stated

“*…I don't think just because the word ‘aggression’ is used in the category name, that is a good rationale to distinguish it from resource guarding - which can also include threats and/or aggression.”*

Among participants, a division on the definition of aggression was evident which seemed to contribute to the disagreement respondents expressed in relation to using the terms *resource guarding* and *possessive aggression*. Generally, participants' discussion of the definition of aggression were divided into two categories: (1) actions that harm or intend to harm the other participant, or (2) threats and harmful actions that primarily serve to increase distance between themselves and the other participant.

Respondents participating in the latter discussion expressed concern about defining and using the word aggression to only mean “intent to harm” as it implies planning on the part of the dog. One individual acknowledged and responded to this concern by stating,

“*Obviously we cannot 100% know an animal's intent, I use it as ‘obvious intent’ which includes anything from lip lift and growl to bite.”*

When asked to provide specific examples of what distinguishes between threats and harmful behavior from another participant, responding individuals generally agreed that “growling, teeth baring, and freezing” are examples of threatening behavior, whereas “snapping, lunging, and biting” are all examples of harmful actions. Two participants admitted that the line between threatening and harmful behavior is not always distinguishable; aggression lies on a continuum and the dog's ultimate response will be a result of the dogs' experience, genetics, and the behavior of the other individual. To validate the two group's contradictory opinions on the definition of aggression, one participant cited numerous quotes from the literature that reflected a similar, long standing level of disagreement on the definition of the word “aggression” within the scientific literature. These quotes included:

“the term aggression has so many meanings and connotations that in effect it has lost its meaning…it is not a simple unitary concept and therefore cannot be defined as such (25).”, “The word ‘aggression’ is widely used and misused in a variety of contexts (26),” and, “The solution to the problem of ‘aggression’ is simply to treat the word as a convenient, loosely defined aid to communication…recognizing that we cannot provide an adequate definition and that we are probably lumping together a number of diverse phenomena (27).”

### Concern for owner perception

In addition, several respondents reported that a client's interpretation of the words used in a term and their concern for owner perception of the behavior influenced their term choice. Concern focused on the potential for the owner misinterpreting the dog's motivation to perform the behavior, and ultimately were about the reaction of the owner. The term *possessive aggression* was discussed most often, with some specifically mentioning concern with the word “possession” and others with the word “aggression.” According to three participants, the word “possession” may be interpreted by the owner as an indication that the dog is vying for inappropriate ownership of the item resulting in a necessary competition between the owner and dog for the item. Further, concern for the word “aggression” focused on misunderstanding of the motivation for the behavior, resulting in an attempt for the owner to control the dog's actions. For example, one participant stated,

“*this terminology…may be deleterious in that it may contribute to inappropriate treatment of aggression problems (combative/confrontational techniques/punishment).”*

And another stated,

“*I think that the term ‘possession’ may have misleading meanings (the same way the term ‘dominance’ does) and may induce the owner to engage in dangerous confrontations with the dog to determine who ‘possesses’ something.”*

Participants suggested that terms that include the word aggression might be avoided because of the negative connotation. For example, one participant stated

“*A lot of people seem to want to use ‘resource guarding’ because it doesn't sound that bad, that if the dog had ‘aggression’ that the dog is a bad dog.”*

Not all respondents felt it necessary to avoid terminology based on its potential perception by owners, even though they recognize that many professional people may do so. For example, one respondent stated

“*Possessive aggression explains the dog is displaying aggression, which can vary in intensity, etc. I don't think the word ‘aggression’ is a bad word, but it is often avoided when some people talk with owners.”*

Almost two-thirds of respondents (10 out of 14) stated their preference for the use of the term *resource guarding* over *possessive aggression* due to the potential for motivation to be interpreted more accurately by owners. For example, one participant stated,

“*I always use resource guarding…the term resource guarding underlies the motivation for the behavior, making the owner understand that the dog is protecting a high value item.”*

These participants mentioned the importance of understanding the motivation for the behavior, both for themselves (treatment and management advice) and for owners. Several participants provided their interpretations of the motivation behind *resource guarding*, which included the following participant definitions: “a resource that the dog is guarding from other individuals being able to obtain,” an “attempt to maintain control/ownership of an item,” and “protection of a high-value item.” Two respondents warned that it is inappropriate to assume motivation, and that motivation may be different for each dog even though the behavior has the same or similar appearance.

### Lay vs. expert communications

The majority of participants (12 out of 14) identified differences in the way they relay information between colleagues (e.g., DVMs or PhDs with behavioral expertise) and clients (i.e., pet owners) with respect to behavior issues. Participants indicated that although they may use strict ethological definitions of aggression when discussing the behavior with colleagues, generally they find themselves adjusting their definitions and language use when discussing the behavior with clients and dog owners to ease communication and attempt to prevent misunderstanding. Participants who consider aggression to include “harmful or harm-intending behaviors only” recognized that most dog owners define aggression more broadly and include behaviors such as growling and teeth baring when discussing aggressive behavior and will adjust their language to mirror that of their clients. For example, one respondent stated

“*with colleagues…I use very specific ethological definitions and descriptions. With clients and DVMs without behavioral expertise, I use aggression in the way that most people outside the field do—any behavior that makes people concerned about a bite or an actual bite. It's not great to use different definitions, but to be practical, I always try to speak the language of the listener.”*

Seven (out of fourteen) participants stated that they use the same terminology with presumed laypersons as they do with experts; however, they take the time to go into more detail with lay individuals when explaining the behavior. For example, one participant stated

“*I would do a lot more explaining to clients (than colleagues), pointing out the details of their dog's behaviors and the context(s) in which they occur but the language would remain the same.”*

For these individuals, the depth of explanation about the behavior relies on the perceived knowledge level of the audience in order to communicate more efficiently. In addition, as discussed previously, a few other participants indicated that they would avoid using certain terms that might have associated negative connotations with clients, such as the word “aggression,” in order to appease clients or avoid misunderstanding.

### Developing a definition

#### Specifying behaviors

In the discussion board, the majority of participants (12 of the 14) suggested changes to the definition proposed for *resource guarding* or *possessive aggression* (Table 3). Half of those participants suggested that the definition should include specific behaviors (e.g., “growling, snapping, biting”) instead of using an inclusive term open to misinterpretation (e.g., “threatening” or “aggressive”). A variety of specific behaviors were proposed, including: body blocking (one out of six), rapid ingestion (two out of six), submissive postures (two out of six), lip lifting (two out of six), grabbing the item and running away (three out of six), stiffening (three out of six), barking (five out of six), growling (six out of six), lunging (six out of six), snapping (six out of six), and biting (six out of six). All suggestions of specific behaviors were either preceded by “for example” or concluded with “etcetera” indicating their proposal represents a sample of the possible behaviors to be included in the behavioral repertoire.

**Table 3:** Agreement with components of a definition gathered from survey stage 1 and presented in stage 2.

**Definition Components**		**# Participants**
Component presented for comment	“Display of threatening or aggressive postures”	28/29 (97%)
	Dog is “in control or perceived control of the object or item”	27/29 (93%)
	“The maintenance or defense of control of the resource”	20/29 (69%)
	“Object or item has some value to the dog”	11/29 (38%)
Participant suggestions	Make description of behaviors or postures more specific	3/29 (10%)
	Include defensive or fearful postures in addition to aggression and threats	1/29 (3%)
	Eliminate term ‘aggression’	1/29 (3%)

#### Defensive vs. offensive

The use of the word “defensive” in the proposed definition received a lot of attention from participants. Five participants discussed the merit of including the word in the primary definition or using it in a modifier, which could be added or subtracted from the definition depending on the body posture of the dog and the context of the behavior. A few of these participants (three out of five) suggested that threatening and aggressive behaviors could be offensive or defensive, and one individual suggested these terms could be applied on a case-by-case basis to help describe the dog's body posture. One individual suggested that the motivation for the behavior is defensive and suggested that the definition include examples of a defensive response:

“*Resource guarding is a behavior motivated by the defense of valuable resources, as perceived by the dog. It can be displayed as a purely defensive response (e.g., running away with valuable items) or as an overtly aggressive response (e.g., growling, barking, lunging and biting).”*

In response, another individual disagreed and stated that the observed behavioral response may be a reflection of past encounters in which the dog was previously punished in a similar scenario and the behavioral response is often more complicated than the defense of a resource. Another participant agreed with the latter and mentioned that motivation for the behavior does not belong in an ethological definition.

#### Affective states

One-third of the 12 participants who suggested changes to the initially proposed definition mentioned the importance of considering underlying affect (e.g., anxiety, as a component of *resource guarding* or *possessive aggression* for some dogs) and adding a subsequent modifier to describe associated body postures such as fearful body postures when anxiety is involved. For example, one participant stated,

“…adding something like, ‘at times fearful, submissive or deferent body postures may be associated with the behavior when anxiety is a component.”

#### Terminology disagreement

Use of the word “valuable” was debated by a few individuals. Two individuals preferred incorporating the word into a definition, suggesting that the item(s) hold some degree of value to the dog based on their unwillingness to relinquish the item. Two other individuals felt that this word is anthropomophic and assumes what the dog might be feeling toward the item or about the situation in general and suggested that use of the word “valuable” is either avoided or preceded by the word “perceived” (e.g., “perceived to be valuable,” or “perceived threat to maintaining possession of the object”).

The word “item” was suggested as a replacement for the term “food or non-food object” by two individuals. Alternatively, two individuals stated that the term “non-food object” and “item” are both too vague and instead they should be described in separate categories as items the dog can physically “take into possession (e.g., pick up in mouth)” or “possess in another way (e.g., lying on the couch or sitting near an individual).” There was some discussion regarding whether resource guarding or possessive aggression is a sub-category of territorial aggression as one participant defined a territory as “any defended area,” and suggested that “people, areas, objects, food, etc.” can all be defended or guarded. Three participants disagreed with this view, with one stating they believe aggression in defense of an area (specified as a “yard, home or car”) or person is not correlated with aggressive behavior around food, objects or sleeping and resting places. Another participant remarked they are unclear about whether people are being “possessed” or “guarded” when a dog behaves with threats or aggression upon approach by another person or other animal. One participant supplied an alternate definition of a territory as,

“*a fixed area from which an individual animal excludes rival intruders by some combination of advertisement, threat and attack (Brown, 1971),” and stated that “territorial behavior involves a whole other set of behaviors that don't belong under the topic of resource guarding (e.g., scent marking).”*

Complicating the development of a definition was the division in agreement over *possessive aggression* and *resource guarding* being synonyms or terms requiring separate definitions. For example, one participant provided separate definitions for *resource guarding* and *possessive aggression*, stating:

“*Resource guarding involves using a variety of behaviors for the purpose of maintaining possession of a particular resource. Possessive aggression is the use of aggressive behaviors in order to maintain possession of a particular resource.”*

Another participant expressed their confusion over the use of separate definitions as they believe the terms are interchangeable and refer to the same pattern of behavior. All suggested modifications to the originally proposed definition are summarized in Table 4.

**Table 4:** Content analysis of agreement and disagreement to the proposed definition by participants.

**Category**	**Behaviors**	**# Agree**	**Target**	**# Agree**	**Underlying Affect or Motivation**	**# Agree**	**Object/Item types**	**# Agree**
Original definition	Defensive, threatening or aggressive	2	Person or other animal	2	Prevention of loss, desire to maintain	4	Food or non-food object	2
Proposed substitutions	“Changes in affect” (used as a blanket term for observed behaviors)	1	Another individual	1	Maintaining control of “*valued”* resource	2	“item” better than non-food object	2
	Agonistic behaviors	2			“*Perceived”* valued resource control	2	Distinction made between items dog can hold in mouth or not hold in mouth (e.g couch)	2
	Avoidance (instead of defensive)	1			“*Perceived”* potential loss to possession	1		
Proposed additions	Tense body posture	1	N/A		Defense (as the motivation)	1	People	2
	Specific behaviors (e.g., taking item and running off, rapidly consuming item, stiffening and hovering over it, lip lifting, barking, growling, lunging, snapping, biting)	6					Resting and sleeping places (e.g., couches)	2
							Territories (i.e., “area”)	1
Proposed Context-specific inclusions	Anxious behaviors (e.g., lip licking, yawning, averting gaze, freezing)	2	N/A		Anxiety (as a component)	2	N/A	
	Defensive or offensive, depending on behaviors observed	3						
	Deferent or submissive	1						
Proposed deletions	Defensive should not be included	2			Should not include motivation in a definition	1	People and territories should not be included	2

*The original proposed definition was: “A dog that is displaying defensive, threatening, or aggressive behaviors to prevent a person or other animal from gaining access to a food or non-food object.” The “number agree” represents a count of individuals that either proposed the respective component or agreed with a previous participant regarding the component proposed (N = 14)*.

## Discussion

### Terminology preferences

Based on the survey, participants preferred the term resource guarding over possessive aggression, and there was no effect of professional qualification on this preference. Participants on the discussion board expressed a variety of perspectives about the use and meaning of the terms *resource guarding* and *possessive aggression*. Most experts initially expressed the belief that the terms *possessive aggression* and *resource guarding* described two different behavior patterns. However, upon analyzing the experts' discussion it became clear that the majority of experts believe *possessive aggression* describes a more specific sub-set of the behavior pattern and is limited to the expression of aggressive behaviors. Individuals participating in this discussion preferred the term *resource guarding* over *possessive aggression* for a variety of reasons including a broader inclusion of behaviors, a greater potential for dog owner understanding of the motivation and relatively positive dog owner perception of the behavior [see (3) for a contradictory argument].

A number of participants expressed the belief that dogs use strategies other than aggression to maintain control of valuable items in support of their preference for the term *resource guarding*. Examples mentioned include grabbing an item and running off with it and rapid ingestion of an item (i.e., avoidance strategies). These types of non-aggressive strategies are rarely mentioned in the literature when describing the behavioral response. Perhaps these behaviors are of less concern to owners (and clinicians) due to their relatively low risk of injury, and thus are seldom presented to a behavior specialist and not widely considered in text book descriptions of *resource guarding* and *possessive aggression*. This focus on aggression may be why the term *possessive aggression* has been used more frequently in reference manuals than *resource guarding*, and the description of the behavior almost always includes the use of aggression [e.g., (3, 10)].

Although anecdotal, experts suggested that many dog owners react negatively to the term *possessive aggression* due to the owner's perception of both words within the term (28). One of the topics that received the most discussion was whether the word “aggression” unduly influences the meaning of the term for owners, inhibiting their understanding that avoidance behaviors can also be displayed and that the behavior is often normal (29). In a focus group described in Orrit et al. (30), professionals reported their biggest challenge was battling the stereotypes and misconceptions with owners of dogs that commonly display aggressive behavior, while in a separate but complimentary focus group, lay persons were defensive when discussing aggressive behavior from their own dogs; these contradictory perspectives likely challenge communication between the two groups. Further, previous research suggests that dog owners and lay persons may have more difficulty identifying or describing aggressive behavior compared to professionals (4, 5, 31), further exacerbating communication issues. The findings from these studies support those observed from our population of participants and suggest that concerns regarding the owner's interpretation of words used in a term have merit.

Furthermore, there was an underlying concern among participants about owner perception of the behavior when the word “possessive” or “possession” was applied, fearing that owners might misinterpret the behavior as a competition with the dog over item ownership, potentially leading to the application of positive punishment-based training methods. These concerns seem to parallel some of the discussion surrounding the labeling of a “dominant” dog. Historically, confrontational techniques have often been employed to make a “dominant dog” submit to the owner or to reinforce the dog's dominant position to other household dogs (32). Research suggests that the application of confrontational training techniques (e.g., using a shock collar or hitting) is associated with and can exacerbate aggressive behavior [e.g., (33, 34)]. Recent position statements from a variety of veterinary organizations disagree with these practices (e.g., American and Canadian Veterinary Medical Associations, Australian Veterinary Association, American Veterinary Society of Animal Behavior) and it seems reasonable to be concerned about the impression of any word that might inadvertently imply the need for confrontational techniques.

Concern over term perception likely contributed to several participants' belief that the term *resource guarding* better communicates the motivation of the behavior, with the aim of promoting owner understanding about the normal nature of the behavior and ultimately decreasing the chance that performance might result in conflict between the owner and the dog. Several of these participants provided their interpretation of *resource guarding* motivation. These suggestions had only slight variation between them and were generally described as “guarding,” “protection,” or “control” over high-valued items. Several other participants were displeased with these suggestions, stating that motivation cannot be known or at the very least, generalized to all dogs exhibiting the behavior. It appeared that these individuals were concerned with knowing the motivation beyond the desire to maintain access to a resource (i.e., hunger, primary access).

One area that did not have much variation in response involved discussion around the use of the term *food-related aggression*. This term has been used throughout the literature to describe an aggressive behavioral response toward a person or other animal when a dog has a food item [e.g., (3, 35)]. The vast majority of participants believe that using a separate term to describe *resource guarding* and *possessive aggression* around food is unnecessary and further complicates the issue. Participants mentioned that food is often narrowly interpreted by humans and that dogs may find items edible that we would not consider to be food. This is an interesting point and one that challenges the literature referenced by another participant that suggests there is different brain circuitry involved in mediating *food-related aggression* from other types of aggression. The cited reference does suggest there are two distinct neural circuits that mediate two different kinds of aggression (in rats and cats), “defensive rage” and “predatory attack” (24). It is unclear which of these two types of aggression the participant was comparing to *food-related aggression* although one might assume predatory attack as it is the natural method of obtaining food (even though predation is directed at the food item, rather than a potential food stealer). Although predation has been listed as one category of aggression by a few authors (36, 37), Archer (29) argues there are only two broad functional types of aggression: resource competition and reactions to danger. Neither of these involve predation.

### Developing a definition

Ethology studies generally focus on the following four main areas, or questions, as proposed by Tinbergen (38): evolutionary history, development, causation and function. The apparent function of a behavior (i.e., the consequences of the behavior) is often included in operational definitions of animal behavior (39). For example, Broom and Fraser (40) define competition as “the striving of two or more individuals to obtain a resource that is in limited supply.” In this example, the immediate function of the behavior is the resulting access to a limited resource. The apparent function of *resource guarding* or *possessive aggression* is the control of an item. Given that the consequence of the behavior pattern is likely to be the same regardless of the specific behaviors that occur (e.g., running away with an item vs. snapping or biting) a complete description of the behavior pattern should allow for extension of the definition beyond aggression alone. In a clinical setting, it might be advantageous to distinguish between behaviors that have been displayed in different contexts in order to assess and mitigate future risk of aggression; however, we propose that the operational definition be inclusive of those behaviors that support the apparent function of the behavior pattern.

Although half of participants preferred the inclusion of specific behavior examples in the definition, the use of a finite list of examples in this context is likely to be too rigid and, therefore, limiting. It is possible that conflicting behavior patterns or those not commonly observed may be used by the dog for the same functional purpose of retaining control of an item; thereby the dog's behavior pattern is functionally representative of *resource guarding* or *possessive aggression* but would not be labeled as such based on the absence of key behaviors. Furthermore, an ethogram for this behavior pattern does not currently exist in the scientific literature, so there is insufficient data on which to base inclusion of specific behaviors in the definition. Indeed, when participants proposed specific behaviors for inclusion in the definition they either preceded their list with “for example” or concluded their list with “etcetera,” which indicates the list was not exhaustive. For these reasons, we propose to include only broad terms in the general definition at this time. As more definitive research is completed, the definition may be augmented with an ethogram that further defines the specific behaviors that relate to each of these broad terms.

One area that received much attention by participants was the discussion around inclusion of the words “defense” or “defensive” in the definition. Participants seemed to be using these words in two different ways, with one group considering “defense of the resource” as a motivation for the behavior, and the other group considering “defensive” as a way to describe the behavioral response (i.e., “defensive body postures”). The latter group debated whether the behavior pattern could also appear offensive, with the majority of participants agreeing that aggressive behavior could be either offensive or defensive. It was suggested that it might be important to identify which was occurring through the use of a modifier in the definition in order to infer the emotional state in the dog. Historically, offensive and defensive aggression in animals have been distinguished by attack patterns and bite locations [e.g., (41)], and are suggested to have different situational determinants, emotional and motivational states (42) and therefore differing associated body postures. However, no scientific studies to date have assessed the emotional states of dogs in the context of *resource guarding* or *possessive aggression*, so it is not possible to infer emotional states in this context at this time. In addition, when put into practice, these modifiers would need to be added to a definition post-response by the dog, and would require direct observation to distinguish between offensive and defensive behaviors or would rely on the ability of the assessor, often likely to be the dog owner, to accurately describe or identify body postures. Furthermore, some behavioral responses may be ambiguous, including both offensive and defensive components. For these reasons, we felt the terms defensive and offensive did not belong in a definition of the behavior.

One of the biggest challenges in developing a definition that is likely to be widely acceptable to the canine behavior community revolves around determining which items, objects, areas and individuals should be included. Participants in the current study were varied in their preferences with suggestions to replace “food or non-food object” with the word “item,” to include a separate indication for food, to limit items to those that can be physically held [in the mouth], and to include larger things such as resting areas or people. However, each change was only suggested by one or two people. The literature follows with similar variation. Some authors are non-specific in their descriptions; for example, “food or other resources” (9, 43) and “food” (for *food-related aggression*) and “non-gustatory items” (for *possessive aggression*) (3). Other authors have shown varying degrees of specificity, including: “food, rawhide or toys” (8), “territory, owners or other animals” (44), “food bowls, chew toys, people, pets, or places” (12), and “food bowls, rawhides, real bones, stolen or found items, toys and stolen objects” (10). It seems unclear in both the literature and within participant discussions whether the behaviors of dogs around people and large areas are serving a similar function as behaviors around small objects that can be manipulated. Alternate interpretations (e.g., fear-related, territorial) may suggest the motivation or function of those behavior patterns differ from those employed to retain control of relatively small food and non-food items. This is a topic in need of further scientific investigation, therefore we opted to exclude people and areas from the current definition. With further evidence to the contrary, the definition should be modified.

### Limitations

A range of opinions on the topic were gathered and analyzed in this study. Due to the nature of the discussion board it was difficult to obtain depth from all participants; some individuals provided deeper opinions and a greater number of comments than others as a result of visiting the discussion board more frequently than their peers. However, the choice of a discussion board was preferred in comparison to a focus group because it allowed for anonymity between a group of participants that are likely to know of each other due to their membership in a relatively small pool of professionals, and it allowed individuals to participate regardless of location or potential scheduling conflicts. The results may not reflect the opinions of all experts in the field of canine behavior. Considering there are ~60 DACVB Veterinarians and 30 CAABs in North America we achieved representation from ~16% of this particular population for the discussion board. However, we had greater representation for the initial survey stages that were used to inform this discussion, and for the assessment of term preference. Furthermore, it is unknown if all participants recognized English as their first language; differences in word use and understanding may have impaired some discussions, and regional differences may have influenced preferences in terminology. However, the unique nature of the online discussion board format allowed participants to express themselves freely and anonymously, while qualitative descriptive analysis allowed for exploration on areas of agreement and disagreement amongst experts on components of a definition and terminology used to refer to a relatively common behavior “problem” that would not have been obtained through other research methods.

## Conclusion

As Overall (14) states, “If what we call something affects the way we think about it—and it does—then what we call it is essential.” The majority of participants for both the survey and discussion board indicated that they prefer the term *resource guarding* rather than *possessive aggression*, and the discussion board analysis suggests that many participants find that the term *resource guarding* is less likely to be negatively misinterpreted by dog owners, is easier to communicate to dog owners, and better represents the potential for behaviors other than aggression to be exhibited during the behavioral sequence (e.g., avoidance-related behaviors). Based on discussion among participants, we propose the following basic definition for *resource guarding*: “The use of avoidance, threatening, or aggressive behaviors by a dog to retain control of food or non-food items in the presence of a person or other animal.” As more scientific research is conducted on this topic we welcome modifications and expansion to our proposed definition. Employing consistent definitions and terminology when referencing this behavior pattern will help ensure consistency and progress for future research and will help to avoid confusion between clinicians and clients.

## Ethics statement

Participants were provided with a consent form prior to beginning the study which outlined the aims and methods of the study, as well as any potential risks, and were provided with contact information for the authors should they have any questions. Participants were asked to read the consent form and acknowledge agreement, after which access to the surveys were allowed. All participants were selected by the authors due to their expertise and advanced degrees; therefore no vulnerable populations were included in the study.

## Author contributions

The idea for the paper was conceived by JJ, JC, LN, TW, and DP. The experiments were designed by JJ, JC, and LN. The experiments were performed by JJ, JC, and LN. The data were analyzed by JJ and JC. The paper was written by JJ, JC, LN, TW, and DP.

### Conflict of interest statement

The authors declare that the research was conducted in the absence of any commercial or financial relationships that could be construed as a potential conflict of interest.
